# Genomic sequencing and analyses of HearMNPV—a new Multinucleocapsid nucleopolyhedrovirus isolated from *Helicoverpa armigera*

**DOI:** 10.1186/1743-422X-9-168

**Published:** 2012-08-22

**Authors:** Ping Tang, Huan Zhang, Yinü Li, Bin Han, Guozeng Wang, Qilian Qin, Zhifang Zhang

**Affiliations:** 1 Biotechnology Research Institute, Chinese Academy of Agricultural Sciences, Beijing, 100081, China; 2 School of Biology and Chemical Engineering, Jiangsu University of Science and Technology, Jiangsu, 212018, China; 3 State Key Laboratory of Integrated Management of Pest Insects and Rodents, Institute of Zoology, Chinese Academy of Sciences, Beijing, 100101, China

**Keywords:** Baculovirus, *Helicoverpa armigera*, Multinucleocapsid nucleopolyhedrovirus, Genome sequence comparison

## Abstract

**Background:**

HearMNPV, a nucleopolyhedrovirus (NPV), which infects the cotton bollworm, *Helicoverpa armigera*, comprises multiple rod-shaped nucleocapsids in virion(as detected by electron microscopy). HearMNPV shows a different host range compared with *H. armigera* single-nucleocapsid NPV (HearSNPV). To better understand HearMNPV, the HearMNPV genome was sequenced and analyzed.

**Methods:**

The morphology of HearMNPV was observed by electron microscope. The qPCR was used to determine the replication kinetics of HearMNPV infectious for *H. armigera in vivo*. A random genomic library of HearMNPV was constructed according to the “partial filling-in” method, the sequence and organization of the HearMNPV genome was analyzed and compared with sequence data from other baculoviruses.

**Results:**

Real time qPCR showed that HearMNPV DNA replication included a decreasing phase, latent phase, exponential phase, and a stationary phase during infection of *H. armigera*. The HearMNPV genome consists of 154,196 base pairs, with a G + C content of 40.07%. 162 putative ORFs were detected in the HearMNPV genome, which represented 90.16% of the genome. The remaining 9.84% constitute four homologous regions and other non-coding regions. The gene content and gene arrangement in HearMNPV were most similar to those of *Mamestra configurata* NPV-B (MacoNPV-B), but was different to HearSNPV. Comparison of the genome of HearMNPV and MacoNPV-B suggested that HearMNPV has a deletion of a 5.4-kb fragment containing five ORFs. In addition, HearMNPV *orf66, bro* genes, and *hr*s are different to the corresponding parts of the MacoNPV-B genome.

**Conclusions:**

HearMNPV can replicate *in vivo* in *H. armigera* and *in vitro*, and is a new NPV isolate distinguished from HearSNPV. HearMNPV is most closely related to MacoNPV-B, but has a distinct genomic structure, content, and organization.

## Background

Members of the family Baculoviridae are rod-shaped viruses with circular, covalently closed, double-stranded DNA genomes [1]. This family includes four genera: *Alphabaculovirus* (lepidopteran-specific nucleopolyhedroviruses (NPVs)), *Betabaculovirus* (lepidopteran-specific granuloviruses), *Gammabaculovirus* (hymenopteran-specific NPVs) and *Deltabaculovirus* (dipteran-specific NPVs) [2]. To date, 54 baculovirus genomes have been sequenced ( http://www.ncbi.nlm.nih.gov/genomes/GenomesGroup.cgi?taxid=10442), including 37 from *Alphabaculovirus*, 13 from *Betabaculovirus*, three from *Gammabaculovirus* and one from *Deltabaculovirus*. Nucleopolyhedrovirus (NPV) and granulovirus (GV) are distinguished from each other by their occlusion body morphology. The NPVs produce large, polyhedron-shaped occlusion bodies, called polyhedra, which contain many virions, whereas the GVs have smaller occlusion bodies, called granules, which normally contain a single virion. The NPVs are further designated as single-nucleocapsid (S) or multinucleocapsid (M), depending on the potential number of nucleocapsids packaged in an envelope of the virion.

The cotton bollworm, *H. armigera*, is a serious pest that causes economic losses to over 60 vegetable and field crops throughout the world [3]. *H. armigera* larvae are significantly resistant to chemical insecticides; therefore, baculovirus pesticides have been recognized as one of the most promising agents to control such pests [4]. HzSNPV was registered as one of the first commercial baculovirus pesticides (Virion-H, Biocontrol-VHZ, Elcar) in the 1970s, and has been used extensively to control the cotton bollworm in the USA [5]. HearSNPV was also the first commercial baculovirus pesticide used to control *H. armigera* in China, and has been extensively used for the control of the pests in vegetable crops [6].

The DNA genomes of HearSNPV-G4 [7], HearSNPV-C1 [8], HearNPV-NNg1 [9], and HzSNPV [10] have been sequenced. Among them, the HearSNPV-G4 and HearSNPV-C1 were isolated from China, HearNPV-NNg1was isolated from Kenya, and HzSNPV was isolated from the United States. Comparative genomic analyses showed that overall gene content and arrangement in these four viruses were highly conserved, and they are considered variants of the same NPV species [9]. In addition, the nucleotide sequence of the HearGV DNA genome was reported [11]. Multinucleocapsid NPVs isolated from *H. armigera* (HearMNPV) producing ODV virions with multiple nucleocapsids per envelope have been identified [12,13]. The genes of other 18 HearMNPV isolates from *H. armigera* which included *lef-8*, *lef-9, polyhedrin* have been reported [14].

In this study, a new nucleopolyhedrovirus isolated from *H.armigera* was observed by electron microscope (EM), suggesting it was multinucleocapsid NPV. Experimental infection of insect larvae indicated that host range of HearMNPV was different from that of HearSNPV and that the cytopathological effect of HearMNPV differed from that of HearSNPV. This report describes the sequence and organization of the HearMNPV genome and compares it with sequence data from other baculoviruses, such as HearSNPV and MacoNPV-B.

## Methods

### Viruses and insects

HearMNPV was originally isolated from a naturally infected *H. armigera* in the Shanghai city, China in the 1970s. The virus was propagated in laboratory stocks of healthy third instar *H. armigera* larvae by *per os* infection. A laboratory stock of eastern armyworm, cotton leaf worm and beet armyworm were reared at 26°C with a 16:8 h light:dark cycle on a semi-synthetic diet.

### Virus purification, DNA extraction, and construction of genomic DNA libraries

To generate a large number of polyhedra, healthy third instar *H. armigera* larvae were inoculated and the hemolymph was collected from the *H. armigera* larvae were collected on ice and centrifuged for 10 min at 4°C. The precipitate was washed several times with distilled water and re-suspended in 0.1% SDS for 30 min at room temperature. After centrifugation, the clean polyhedra were re-suspended in 200 μl TE buffer (10 mM Tris–HCl, pH 8.0, 1 mM EDTA) [15].

The genomic DNA of HearMNPV was purified according to the following protocol: about 5 × 10^8^ polyhedra were dissolved in 0.1 M Na_2_CO_3_, 0.15 M NaCl, pH10.4 on ice for 10 minutes, SDS was then added to a final concentration of 0.5%, and the solution was kept on the ice for another 10 minutes. The genomic DNA was extracted twice in an equal volume phenol (pH8.0) and once in chloroform. The DNA was precipitated with two volumes ethanol, washed with 70% ethanol, and dissolved in 0.1 × TE buffer (pH8.0) [16]. The quantity and quality of the isolated DNA were determined by spectrophotometrically and by electrophoresis on 0.7% agarose.

A random genomic library of HearMNPV was constructed according to the “partial filling-in” method and contained 2.0 to 5.0 kbp fragment in vector pUC19 [15,16] DNA fragments for sequencing were prepared from 527 recombinant plasmids. The recombinant plasmids were sequenced with plasmid specific primers and 'primer nesting' from both strands, using BigDye Terminator v3.1 (ABI) on a 3130XL Genetic analyzer (ABI). The combined sequence was generated from these clones represented a six-fold genomic coverage. The gaps were filled by PCR.

### The insect cell lines and infection

The Hz-AM1 cell line and HaBacHZ8-GFP were gifts from Dr. Fei Deng of Wuhan Institute of Virology, Chinese Academy of Sciences. HaBacHZ8 is a bacmid of HearNPV that lacks the *polyhedrin* gene. An enhanced GFP gene was introduced to HaBacHZ8 by using the HearSNPV bac-to-bac system [17,18] and this generated the bacmid HaBacHZ8–GFP [19]. The QB-Ha-E-5 cell line, which was a gift from Dr. Guiling Zheng of Shandong Agricultural University, was established from the embryonic tissue of *H. armigera* (Lepidoptera: Noctuidae). The cell line had been subcultured over 60 passages in TNM-FH medium supplemented with 10% fetal bovine serum. The cell line could be infected by *H. arigera* single nucleopolyhedrovirus (HaSNPV) [20]. The Hz-AM1 cells and QB-Ha-E-5 cells were cultured at 27°C in TNM-FH insect medium (Sigma, USA) supplemented with 10% (v/v) heat-inactivated fetal bovine serum (Gibco-BRL, Gaithersburg, USA). Hz-AM1cells and QB-Ha-E-5 cells were infected with HearMNPV at a multiplicity of infection (MOI) of 5. For coinfection, QB-Ha-E-5 cells were infected simultaneously with HearMNPV and HaBacHZ8-GFP at an MOI of 5 for each virus. The cells were examined using Nikon-Ts100 and Leica TCS SP5 II microscopes.

### Scanning electron microscopy

Polyhedra were fixed in 2.5% glutaraldehyde at 4°C for 2 h. The fixed sample was dehydrated through a serial ethanol gradient, and then embedded in Epon-Araldite resin. A diamond knife was used to cut ultrathin sections on a Reichert OMU3 Ultramicrotome. The sections were stained with 2% aqueous uranyl acetate, followed by lead citrate. Micrographs of the Polyhedra were taken with a Hitachi S3400N transmission electron microscope at 80 kV.

### Transmission electron microscopy

Polyhedra were fixed in 2.5% glutaraldehyde in 0.05 cacodylate buffer at 4°C for 2 h and post-fixed in 1% osmium tetroxide in the same buffer for 2 h at room temperature. Fixed samples were dehydrated through a graded series of ethanol solutions and embedded in Spurr’s resin. Sections were cut, stained with uranyl acetate and lead citrate, and examined under a JEM-1230 transmission electron microscope (TEM) at an accelerating voltage of 80 kV.

### Quantitative PCR (qPCR)

Third-instar larvae were starved for 12 h at 26°C before being inoculated, and *H. armigera* test larvae were allowed to ingest a diet soaked in a 10 μl drop, containing an estimated 10^7^ OBs. Control larvae ingested a diet soaked in a 10 μl drop with no OBs. The diet soaked OBs or ddH_2_O were replaced by fresh diet with no OBs after 2 hours adsorption period at 26°C. Time zero of the infection was defined as the time when the diet soaked OBs or ddH_2_O was removed from the culture boxes. Larvae used in this experiment were sacrificed at various time points ranging from 4 to 96 h post-inoculation (p.i.). A powder was prepared from ten larvae using a mortar and pestle after each collection under liquid nitrogen. 0.1MNa_2_CO_3_, 0.15 M NaCl, 1%NP-40 was added to the powders to a total volume of 700 μl. Total DNA was then extracted by the addition of an equal volume of phenol (pH8.0) (twice) and chloroform (once). The DNA was precipitated with two volumes of ethanol, washed with 70% ethanol, and dissolved in ddH_2_O. The quantity and quality of the isolated DNAs were determined spectrophotometrically.

HearMNPV DNA copy number was determined by real-time qPCR with primers specific to the *rr2b* gene. The viral copy number was then normalized against host-genome copy number by qPCR with primers specific to the host actin gene [21]. The *rr2b* and *actin* genes were amplified by PCR and cloned into pGEM-T. Recombinant plasmid DNA concentrations were quantified using a spectrophotometer and dilution standards were generated. For each standard dilution, three independent qPCRs were performed using *rr2b* or *actin* specific primers, and standard curves were generated. For each larval DNA extract, three independent qPCRs were performed using *rr2b* and *actin* specific primers. The mean of the HearMNPV DNA copy numbers were determined and the number of *rr2b* amplicons was normalized against the number of host *actin* genes to derive the mean number of viral copies per mean host actin gene copy number. The specific primers were as follows:

*actin*-F 5' CTCTTCCAGCCCTCATTCTTG 3'

*actin* -R 5' TTCTGCATACGGTCAGCGATA 3'

*rr2b*-F 5' AGCAACAAGACTTAATACTCAACGC 3'

*rr2b*-R 5' AATATGGCTGCAAAGCTACCG 3'

### DNA sequence analysis

Restriction fragments from recombinant plasmids were sequenced and assembled into contigs using SeqMan5.0 from the DNASTAR software package. PCR was used to generate gap-spanning fragments and low quality data regions after preliminary assembly. Open reading frames (ORFs) were identified using ORF Finder http://www.ncbi.nlm.nih.gov/gorf/gorf.html[22]. The criterion for defining an ORF was a size of at least 150 nt (50 aa) with minimal overlap. Promoter motifs present upstream of the putative ORFs were screened as described previously [23]. Homology searches were done through the National Centre for Biotechnology Information (NCBI) website using BLAST [24]. Multiple alignments and percentage identities were performed using the Clustal W. The Tandem Repeats Finder http://tandem.bu.edu/trf/trf.html was used to locate and analyze the homologous regions (*hr*s) [25]. GeneParityPlot analysis was performed as described previously [26]. A phylogenetic tree was inferred from amino acid sequences by NJ and MP analyses using MEGA, version 5.0 [27]. Bootstrap analyses were performed to evaluate the robustness of the phylogenies using 1000 replicates for both NJ and MP analyses.

## Results and discussion

### Electron microscopy observation

**Figure 1:**
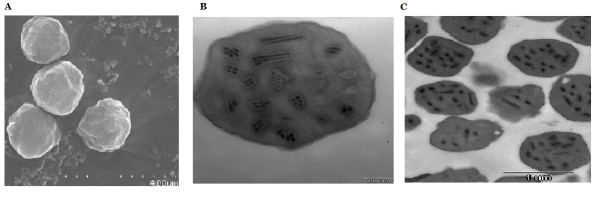
**Electron micrographs of polyhedra from HearMNPV and HearSNPV.A**. Scanning electron micrograph of HearMNPV (12,000×) **B**. Transmission electron micrograph of HearMNPV (80,000×) **C**. Transmission electron micrograph of HearSNPV (20,000×).

Scanning electron microscopy revealed that the purified occlusion bodies (OBs) of NPV originating from infected cotton bollworm have irregular shapes, with diameters of about 2 ± 0.3 μm (Figure 1A). Transmission electron microscopy showed multiple rod-shaped nucleocapsids of about 230 nm in length and 50 nm in width embedded in each OB, with multiple nucleocapsids packaged within the envelope of the virion (Figure 1B). These results indicated that the virus is a typical multinucleocapsid NPV. However, transmission electron microscopy indicated that HearSNPV have single nucleocapsids packaged in their virion (Figure 1C). Therefore, the isolate was termed *H. armigera* multinucleocapsid nucleopolyhedrovirus (HearMNPV)*.*

### HearMNPV infected insect larvae and cells

**Figure 2:**
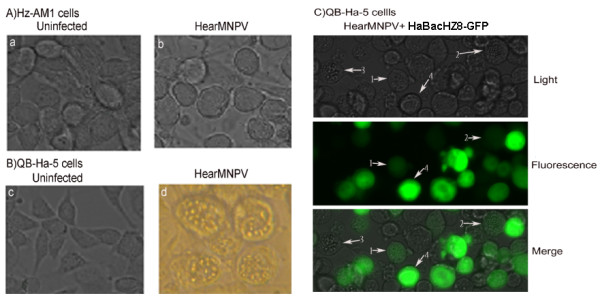
**Cytopathic effects in infected cells.** Panel **A**: Hz-AM1 cells uninfected (A) and infected with HearMNPV (MOI:5) at 72 hpi (B). Panel **B**: QB-Ha-E-5 cells uninfected (C) and infected with HearMNPV (MOI:5) at 72 hpi (D). Panel **C**: QB-Ha-E-5 cells co-infected with HearMNPV and HaBacHZ8-GFP (MOI:5) at 72 hpi. Cells were viewed using confocal laser fluorescence microscopy. Arrows 1 and 2 indicate polyhedra-containing fluorescent cells, arrow 3 indicates polyhedra-containing cells, and arrow 4 indicates fluorescent cells. Magnification: ×400.

Experimental infection of insect larvae showed that HearMNPV can infect the eastern armyworm (*Pseudaletia separate*), but cannot infect either the cotton leaf worm (*Spodoptera litura*) or the beet armyworm (*Spodoptera exigua*). By contrast, HearSNPV cannot infect *P. separate*. These results indicate that the host range of HearMNPV differs from that of HearSNPV. Moreover, HearMNPV-infected Hz-AM1 cells produced no polyhedra and showed no typical cytopathic effects (CPE), even at 96 h post-infection (pi) (Figure 2A). However, HearMNPV-infected QB-Ha-E-5 cells produced polyhedra (Figure 2B). It was previously reported that Hz-AM1 cells were permissive to HearSNPV-G4 [8,17,18]. When QB-Ha-E-5 cells were infected by HaBacHZ8-GFP [18,19], which was constructed from HearSNPV, green fluorescence was observed under fluorescence microscopy (Figure 2C). These results indicate that the host range and cells infected by HearMNPV differ from those infected by HearSNPV.

Coinfection of QB-Ha-E-5 cells with HaBacHZ8-GFP and HearMNPV showed that certain cells possessed green fluorescence (under fluorescence microscopy), some cells produced polyhedra, and some cells possessed both green fluorescence and polyhedra (Figure 2C). The results indicate in cells coinfected with two distinct viruses, the viruses are able to coexist, replicate, and package themselves independently. *Cydia pomonella* granulovirus (CpGV) is one of the most successful commercial baculovirus insecticides; however, resistance of the codling moth (*C. pomonella*) to commercially applied CpGV in orchards located in Germany and France has occurred [28]. Therefore, alternating virus treatment or using a mix of HearMNPV and HearSNPV could delay the development of resistance in *H. armigera*, helping to improve both the prevention and control of *H. armigera* in the field.

### HearMNPV virus DNA replication *in vivo*

**Figure 3:**
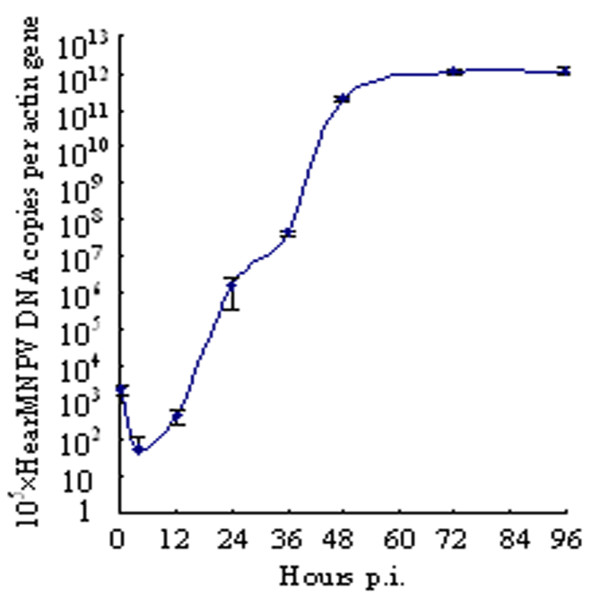
**Quantification of HearMNPV DNA copy number in infected *H. armigera *larvae.** qPCR was used to determine the number of *rr2b* gene copies relative to the actin gene at various times following infection. The plotted points indicate the averages of the number *rr2b* gene copies relative to actin gene (performed in triplicate).Error bars represent standard deviations.

HearMNPV is a potentially new isolate infectious for *H. armigera* based on the analysis of host range and morphology. Thus qPCR was used to determine the replication efficiency of HearMNPV infectious for *H. armigera in vivo*. Figure 3 shows the quantity of HearMNPV viral DNA in infected larvae, in the decreasing phase (0–4 hr), latent phase (4–12 h), exponential phase (12–48 h), and stationary phase (48–96 h). Initially, the number of viral DNA (vDNA) copies appeared to decrease between 0 h post infection (p.i.) and 4 h p.i. before increasing by 6.97 times between 4 h p.i. and 12 h p.i. The number of vDNA dramatically increased between 12 h p.i. and 48 h p.i., from 452/10^5^ actin to 2.02 × 10^11^/10^5^ actin, an increase of 4.46 × 10^8^ fold. These results indicated that the viral DNA replicated about 29 times, taking about 1.24 h to generate another vDNA copy. This trend continued into the stationary phase, to a lesser degree: vDNA increased 4.82 times between 48 h p.i. and 96 h p.i, and there were about1.17 × 10^12^ copies per 10^5^ actin at 96 h. These results suggested that *H. armigera* could be infected by HearMNPV efficiently and the replication kinetics conformed what has previously been described for other baculoviruses [29].

### Nucleotide sequence of the HearMNPV genome

The HearMNPV genome consists of 154,196 bp (GenBank accession no. NC_011615), which is similar to the genomes of MacoNPV-A (155,060 bp) and MacoNPV-B (158,482 bp). The HearMNPV genome has a G + C content of 40.07%, which is within the 58% (LdMNPV) and 32.7% (ChocGV) range for baculovirus genomes, and is similar to MacoNPV-B (40%), AcMNPV (40.7%), BmNPV (40.4%), and EppoNPV (40.7%). According to the adopted convention, the adenine residue at the translation initiation codon of the *polyhedrin* gene represented the zero point on the HearMNPV physical map, and the *polyhedrin* gene was designated as ORF 1 (Table 1). A total of 162 putative ORFs and four homologous regions (*hrs*) were detected in the HearMNPV genome, using computer-assisted analysis to select ORFs starting from a methionine-initiated codon (ATG) and including at least 50 amino acids (aa) and having a minimal overlap with other ORFs [30,31]. All 162 ORFs are shown in Table 1 by location, orientation, size, and potential baculovirus homologs.

**Table 1:** List of ORFs in HearMNPV and their Homologous ORFs in the MacoNPV-B, MacoNPV-A, HearSNPV(G4), AcMNPV, AgSeNPV and HearGV

**ORF**	**Name**	**Position**^**a**^			**Prm**^**b**^	**length**	**Homologous ORFs [ORF number,amino acid identity(%)]**											
							**MacoNPV-B**		**MacoNPV-A**		**HearSNPV(G4)**		**AcMNPV**		**AgSeNPV**		**HearGV**	
1	*polh*	1	>	741	L	246	1	100	1	98.0	1	87.4	8	89.3	1	89.8	1	55.1
2	*1629*	790	<	2358	L	522	2	95.8	2	72.3	2	26.6	9	27.9	2	33.0	2	16.7
3	*pk1*	2357	>	3175		272	3	99.6	3	87.5	3	48.5	10	38.7	3	58.6	3	34.6
4	*hoar*	3250	<	5478	E	742	4	97.1	4	74.3	4	20.2			4	27.7		
5		5967	>	6533	E	188	5	96.8	5	67.2								
6	*odv-e56*	6626	>	7747	L	373	6	100	6	89.3	15	52.6	148	49.2	6	59.5	14	42.1
7	*me53*	7892	<	8956		354	7	99.7	7	86.2	16	30.9	139	24.6	7	47.4	178	22.1
8	*F protein*	9563	>	11599	L	678	8	99.9	9	90.9	133	36.9	23	22.1	8	44.4	26	29.8
9		11719	<	12678	E	319	9	99.1	10	91.2					9	46.1		
10	*gp16*	12729	<	13016	L	95	10	100	11	97.9	119	30.8	130	35.5	10	65.6		
11	*p24*	13029	<	13718	L	229	11	100	12	92.5	118	50.4	129	37.4	11	63.8	78	25.7
12		13784	>	14095	L	103	12	100	13	91.3	117a	34.6			12	44.4		
13	*lef2*	14049	>	14696		215	13	99.1	14	82.0	117	47.9	6	40.6	13	56.6	33	26.8
14	*xe*	14792	>	15175	E	127	14	99.2	15	85.5							21	22.0
	*hr1*	15251		16435														
15	*endonuclease*	16436	<	16732	L	98	15	99.0	17	93.4			79	44.7			69	40.5
16		16798	>	17397		199	16	98.5	18	87.9			70	23.9	25	27.0		
17		17550	>	18260	E,L	236	117	41.2	19	80.6			151	33.0	103	38.9		
18	*chitinase*	18318	<	20006	L	562	19	99.3	22	97.0	41	63.3	126	67.2	23	82.6	105	60.3
19	*bro-a*	20197	>	21192		331	20	83	24	77.7	60	53.9	2	23.3	50	33.2	101	64.3
20		21257	>	21682	E	141	21	99.3	25	86.5							131	38.9
21		21786	>	22595	E	269	22	98.1	26	78.1							52	40.8
22		22706	<	23347		213	23	95.8	27	91.1	57	25.8			49	21.4	81	34.9
23		23525	>	23839	L	104	24	99.0	29	64.4								
24		23960	<	24595	E	211	25	97.6	30	69.2								
25	*hel2*	25012	<	26379	E	455	26	98.9									147	57.7
26	*he65*	26512	<	28266		584	27	98.5	32	91.7	61	28.6	105	37.1	20	53.3	62	53.4
27	*cathepsin*	28331	>	29356		341	28	100	33	97.1	56	46.7	127	56.9	19	81.3		
28		29353	<	29700	L	115	29	100	34	90.6	125	29.0			18	72.0		
29	*lef1*	29728	>	30375		215	30	99.1	35	95.8	124	48.2	14	40.0	17	64.2	80	36.6
30	*38.7 k*	30375	>	31424	L	349	31	99.7	36	92.6	123	34.1	13	29.5	16	60.3	102	23.4
31	*gp37*	31476	>	32264	L	262	32	99.6	37	95.0	58	62.8	64	58.6	26	72.4	109	43.1
32	*ptp2*	32221	<	32760	L	179	33	98.9	38	92.7					27	51.0		
33	*egt*	32828	>	34414	E	528	34	99.4	39	94.4	126	50.7	15	48.0	28	73.1		
34		34583	>	35119	E	178	35	98.9	40	94.9	127	22.3			29	53.3		
35		35119	>	35766		215	36	98.1	41	90.7	128	28.1	17	30.4	30	45.9		
36		35802	<	38357		851	37	99.4	42	88.5	129	26.5			31	45.4		
37	*chtB2*	38415	>	38855	L	146	38	98.6	43	64.6	83	31.7	146	43.8	148	34.0	107	46.2
38		38886	>	39413	L	175	39	99.4	44	82.8			4	26.7	32	36.8		
39	*pkip*	39434	>	39943	L	169	40	100	45	91.6	130	36.2	24	23.5	33	55.6		
40		39965	<	40306		113	41	100	46	96.5					34	52.7		
41	*arif1*	40312	<	41184		290	42	98.6	47	92.0	131	29.2	21	22.7	35	43.3		
42	*pif2*	40940	>	42199		419	43	99.3	48	95.0	132	69.0	22	59.5	36	74.6	42	51.1
43	*pif1*	42214	>	43803		529	44	99.6	49	93.6	111	43.0	119	48.6	37	61.2	82	36.2
44		43800	>	44045		81	45	100	50	96.3	112	32.9			38	44.3		
45	*fgf*	44080	<	45174		364	46	99.2	51	72.9	113	32.0	32	27.5	39	41.7	176	34.0
46		45210	>	46115		301	47	99.7	53	89.5					40	45.7		
47	*alk-exo*	46157	<	47344	L	395	48	99.8	54	90.1	114	41.4	133	38.8	41	48.6	146	39.4
48		47582	<	47920	L	112	49	100	55	93.6	115	27.0	19	26.7	44	65.1		
49		47919	>	49082	L	387	50	99.7	56	94.6			18	25.8	45	62.3		
50		49121	<	49522		133	51	99.3	57	94.7	122	22.5	132	21.9	46	50.0		
51	*rr2*	49594	>	50535		313	52	99.7	58	94.3					47	72.2		
52		50544	<	51593		349	53	100	59	73.1					103	55.3		
53	*calyx/pep*	51620	<	52597		325	61	100	60	97.5	120	41.6	131	32.3	49	65.7	18	21.0
54		52871	<	53209		112	62	100	62	84.1	110	40.2	117	34.2	51	66.0		
55		53161	<	53523	E	120	63	100	63	90.8	109	35.8			52	53.2		
56		53687	<	54301	E,L	204	64	98.5	65	86.8								
57	*sod*	54366	<	54821		151	65	100	66	96.7	106	75.3	31	73.2	54	85.2	63	53.7
58		54878	>	55243		121	66	100	67	86.8					55	47.3		
59	*pif3*	55269	>	55880	L	203	67	98.0	68	92.6	98	51.8	115	47.1	56	70.0	30	44.5
60		55846	>	56316		156	68	98.1	69	87.7	99	22.7			57	57.5		
61	*pagr*	56365	>	57819	L	484	69	99.4	70	87.4	100	23.5			58	38.0		
62		57842	>	58483	L	213	70	100	71	96.8	101	57.1	106	53.2	59	78.2	45	44.1
63	*nrk1*	58518	<	59627	E	369	71	99.4	72	95.0			33	30.1	60	58.4		
	*hr2*	59758		61523														
64		61569	>	62045	L	158	72	93.7	73	87.3			4	25.6	32	20.2		
65	*dutpase*	62105	>	62449	E	114	73	91.2	74	92.2					62	66.7		
66		62531	>	64309	E,L	592	18	99									53	51.0
67	*bro-b*	64565	>	65572		335	74	77.5	75	79.6	60	47.5	2	23.0	50	32.0	101	54.8
68	*p13*	65621	>	66457	L	278	75	99.6	76	95.3	97	58.4			64	68.5	40	47.4
69	*sprT*	66512	>	67036		174	76	98.9	77	89.7							21	30.7
70	*odv-e66a*	67148	>	69166	E,L	672	77	99.7	78	97.2	96	58.1	46	39.7	125	35.9	150	56.3
71		69163	<	69474	L	103	78	99.0	79	96.1	95	44.7	108	32.8	65	59.6		
72	*odv-ec43*	69484	<	70554	L	356	79	100	80	95.2	94	50.4	109	41.6	66	77.3	48	31.9
73		70538	<	70717		59	80	100	81	98.3	93	50.0	110	36.0	67	73.7	46	50.0
74	*vp80*	70714	<	72360		548	81	99.3	82	83.1	92	24.0	104	22.9	68	37.2		
75	*p45*	72388	>	73521	E,L	377	82	99.7	83	97.9	91	58.7	103	51.1	69	80.7	90	39.5
76	*p12*	73508	>	73816	L	102	83	100	84	89.2	90	42.1	102	32.6	70	51.9		
77	*p40*	73842	>	74936	L	364	84	100	85	92.6	89	53.1	101	43.4	71	69.6	92	21.6
78	*p6.9*	74995	>	75228	L	77	85	100	86	83.3	88	58.9	100	62.0	72	64.2	93	16.7
79	*lef5*	75225	<	76046		273	86	99.6	87	96.3	87	50.3	99	57.4	73	77.6	94	43.0
80	*38 k*	75945	>	76847	L	300	87	99.7	88	96.0	86	53.7	98	44.6	74	73.0	95	40.5
81	*vef*	76886	>	79432	L	848	88	99.3	89	81.3					76	39.5	151	28.4
82	*bro-c*	79437	<	80507		356	89	98.3	90	84.4	105	28.9	2	50.1	77	63.7	117	25.1
83		80591	<	81019		142	90	99.3	91	95.8					78	72.5		
84	*odv-e28*	81052	<	81570		172	91	98.84	92	97.7	85	62.9	96	50.6	79	80.2	96	40.5
85	*helicase*	81527	>	85156	L	1209	92	99.83	93	96.5	84	46.8	95	41.8	80	78.7	97	27.8
86	*odv-e25*	85256	<	85906	L	216	93	100	94	94.9	82	64.5	94	45.1	81	83.9	98	48.0
87		85903	<	86388	L	161	94	100	95	98.8	81	69.7	93	51.0	82	88.4	99	32.9
88		86387	>	87145		252	95	99.6	96	94.8	80	57.5	92	51.4	83	82.5	100	37.9
89		87255	>	87770	E,L	171	96	97.7	97	91.2	77	33.9	142	34.7	84	31.7		
90	*lef4*	87802	<	89166		454	97	99.6	98	91.9	79	48.4	90	44.5	85	66.3	112	35.3
91	*vp39*	89165	>	90154	L	329	98	98.5	99	79.6	78	50.9	89	42.1	86	55.2	113	31.9
92	*cg30*	90237	>	91061	E	274	99	97.8	100	80.8	77	21.2	88	21.7	87	28.6		
93	*91 k*	91117	<	93555	L	812	100	99.3	101	92.9	76	44.4	83	41.5	88	58.4	121	28.3
94	*tlp-20*	93524	>	94111	L	195	101	98.0	102	91.9	75	41.4	82	31.4	89	63.7	122	41.2
95		93936	>	94658	L	240	102	99.2	103	82.8	74	62.3	81	49.5	90	68.3	123	51.5
96	*gp41*	94627	>	95628	L	333	103	100	104	97.9	73	55.7	80	57.1	91	80.4	124	36.0
97		95508	>	95963		151	104	100	105	92.2	72	41.5	78	35.9	92	58.3		
98	*vlf-1*	95965	>	97107	L	380	105	100	106	97.4	71	62.8	77	64.0	93	90.5	126	27.6
99	*ctl*	97104	<	97256	L	50	106	98	107	94.0			3	49.0			130	65.3
100		97329	<	98423	E	364	107	98.1	108	82.7	34	22.7			127	22.5		
101	*p26*	98524	<	99258	E	244	108	100	109	97.1	22	27.9	136	31.6	94	66.0		
102	*iap-2*	99307	<	100053		248	109	99.2	110	87.8	62	43.1	71	32.8	95	57.6	139	27.6
103		100010	<	100825		271	110	98.9	111	92.6	63	48.5	69	45.4	96	67.1		
104		100809	<	101174		121	111	100	112	95.9	64	55.1	68	47.9	97	75.6	137	40.4
105	*lef3*	101173	>	102354		393	112	99.5	113	82.2	65	30.5	67	27.0	98	54.3		
106	*desmoplakin*	102414	<	104675		753	113	99.2	114	79.9	66	24.9	66	22.3	99	29.9	135	38.2
107	*DNA pol*	104674	>	107676		1000	114	99.9	115	94.3	67	59.3	65	45.5	100	72.5	134	38.3
108		107710	<	108099	L	129	115	100	116	99.2	69	40.5	75	26.4	101	84.5		
109		108110	<	108367	L	85	116	100	117	100.0	70	70.6	76	41.9	102	87.1	128	32.9
110		108459	>	109199		246	117	97.2	118	86.0			151	61.5	103	39.0	10	32.9
111		109191	<	109736		181	118	98.9	119	87.9	57	31.8			49	23.6	81	29.5
112		109771	>	110232		153	119	98.7	120	96.1								
113		110287	>	110934	L	215	120	96.7	121	88.8					104	46.6	167	30.4
114	*bro-d*	110975	<	112033		352	121	89.6	122	83.6	105	23.4	2	45.1	123	39.3	117	27.9
115	*bro-e*	112087	<	112776		229	122	97.4	123	76.5	59	25.0	2	34.4	77	28.4	133	34.3
116	*lef9*	112859	<	114352	L	497	123	99.6	124	96.4	55	70.2	62	64.5	105	85.9	140	53.5
117	*fp25*	114430	>	115017	L	195	124	100	125	99.0	53	71.2	61	62.8	106	88.7	141	33.6
118	*p94*	115094	>	117598		834	125	99.4	126	82.5			134	41.7			20	35.8
119	*bro-f*	117622	>	118161	L	179	126	98.8	127	95.0	60	42.3			107	64.0	158	50.9
120	*chaB2*	118194	>	118469	L	91	127	100	128	96.7	52	55.2	60	48.8	108	67.4	103	38.6
121	*chaB1*	118447	>	118956		169	128	94.4	129	87.4	51	39.0	59	49.1	109	53.8		
122		118949	<	119428	E	159	129	98.7	130	95.0	50	40.3	57	37.4	110	59.4		
123		119678	<	119947	L	89	130	100	131	92.1	49	55.6	56	42.9	111	60.0		
124		119889	<	120098		69	131	100	132	95.8	48	49.3	55	42.0	112	71.6		
125	*vp1054*	120224	<	121234	E,L	336	132	100	133	93.2	47	53.2	54	40.3	113	67.3	173	35.0
126	*lef10*	121095	<	121322	L	75	133	98.7	134	93.3	46	47.2	53a	50.7	114	62.7		
127		121282	>	121509	L	75	134	100	135	93.3	45	36.0			115	50.8		
128		121523	>	122509	L	328	135	97.3	136	75.0	44	29.0			116	34.0		
129		122514	<	122987	L	157	136	100	137	94.3	43	57.4	53	51.2	117	66.9	169	28.8
130		122986	>	123489		167	137	100	138	88.6	42	26.5	52	20.8	118	49.1		
	*hr3*	123528		124601														
131	*Iap3*	124860	>	125717	L	285	138	99.3	139	82.5	103	33.2	27	28.0	119	43.9	139	25.5
132	*bjdp*	125756	<	126910		384	139	98.7	140	88.0	39	31.5	51	19.5	120	36.6		
133	*lef8*	126931	>	129567		878	140	99.9	141	97.3	38	68.0	50	60.5	121	78.9	149	50.3
134		129595	<	130059		154	141	98.7	142	82.2					122	29.7		
135		130103	<	130303		66	142	100	143	95.4	37	25.4	43	32.3	124	58.6		
136	*odv-e66b*	130351	<	132342	L	663	143	98.6	144	88.8	96	33.8	46	27.9	125	50.2	150	35.4
137	*p47*	132390	>	133583		397	144	99.8	145	97.2	35	61.8	40	55.2	126	75.6	74	46.6
138		133594	<	134643		349	145	99.1	146	85.6					127	34.0		
	*hr4*	134722		135445														
139		135447	<	135710	E	87												
140		135947	>	136519	E	190	146	98.4	147	81.9					129	30.8		
141	*bv-e31*	136581	>	137285	E,L	234	147	100	148	94.9	33	66.5	38	63.3	130	85.0	77	42.4
142	*lef11*	137210	>	137584	L	124	148	99.2	149	90.2	32	49.0	37	39.1	131	64.6	51	38.1
143	*39 k*	137553	>	138407	L	284	149	99.3	150	92.0	31	30.3	36	33.8	132	49.8	50	30.2
144		138476	<	138673		65	150	98.5	151	86.2					134	37.1		
145	*ubiquitin*	138600	<	138902	L	100	151	100	152	93.9	28	77.6	35	77.9	135	94.7	47	76.6
146		138958	>	139503	L	181	152	98.9	153	93.4	27	50.3	34	35.5	136	63.8		
147		139854	<	140210	L	118	153	99.2	154	94.9	26	36.6	26	32.4	138	61.1		
148	*dbp-2*	140299	>	141279	E	326	154	99.1	155	95.4	25	42.8	25	24.2	139	59.9	87	24.0
149	*lef6*	141285	>	141710	L	141	155	98.6	156	93.7	24	45.7	28	25.5	140	47.1		
150		141751	<	141996		81	156	100	157	97.5			29	41.4	141	75.3		
151	*p26*	142112	>	142912	L	266	157	98.9	158	96.6	22	44.9	136	33.1	142	67.1		
152	*p10*	142951	>	143202	L	83	158	100	159	92.9	21	47.1	137	35.1	143	70.4	5	43.4
153	*p74*	143289	<	145262	L	657	159	99.4	160	94.7	20	55.3	138	52.5	144	65.9	72	39.3
154		145343	>	145594	E,L	83	160	98.8	161	91.8	19	32.1			145	53.0		
155	*ie1*	145631	<	147436		601	161	99.3	162	91.1	14	41.5	147	29.4	146	47.4		
156	*ep23*	147478	>	148053	L	191	162	100	163	95.3	13	31.4	146	33.5	147	63.9	9	30.9
157	*chtB1*	148114	<	148392	L	92	163	100	164	92.4	12	55.4	145	44.2	148	70.7	10	40.7
158	*odv-e27*	148395	<	149231	L	278	164	100	165	97.5	11	59.8	144	51.7	149	90.7	114	27.7
159	*odv-e18*	149270	<	149527	L	85	165	100	166	88.4	10	54.1	143	56.3	150	62.1	11	61.1
160	*p49*	149529	<	150914	L	461	166	100	167	97.4	9	57.4	142	49.2	151	78.0	12	35.9
161	*ie0*	150932	<	151636	L	234	167	99.6	168	95.7	8	42.7	141	30.8	152	64.5		
162	*rr1*	151802	<	154087	E	761	168	99.5	169	93.3					153	62.3		

*Note.*^a^Column 3 indicates ORF location and transcriptional direction on the HearMNPV genome. ^b^Column 4 indicates the presence of early (E) and/or late (L) promoters located upstream of the start codon of each ORF. E indicates a TATA sequence followed by a CAGT or CATT mRNA start site sequence 20–40 nucleotides downstream, within 180 bp upstream of the start codon. L indicates the presence of a (A/T/G) TAAG up to 180 bp upstream of the initiation codon.

HearMNPV ORFs had an average length of 870 bp, with ORF85 (*helicase*) being the largest (3,627 bp) and ORF99 (*ctl*, conotoxin-like protein) being the smallest (150 bp). The 162 predicted ORFs encode 46,677 aa. The total coding sequence and intergenic regions were 139,026 and 15,170 bp, which represented 90.16% and 9.84% of the genome, respectively. The four *hrs* were distributed along the genome, with sizes ranging from 724 to 1,766 bp, and their total sequence was 4,749 bp, accounting for 3.08% of the genome. Thirty-eight ORFs overlapped with adjacent ORFs by between 1 and 244 bp, with a total of 1485 bp.

Of the 162 ORFs identified in HearMNPV, 21 possessed a consensus early promoter motif (a TATA box followed by a CAGT or CATT motif 20 to 40 bp downstream, and up to 180 bp upstream, of the initiation codon). Seventy-one ORFs only contained a late promoter motif ((A/T/G) TAAG up to 180 bp upstream of the initiation codon), and nine had both early and late promoter motifs, which might allow transcription during both the early and late stages of infection. Sixty-one ORFs lacked any recognizable consensus early or late promoter motifs up to 180 bp upstream of the ATG. Eighty-six ORFs (46%) were oriented in a clockwise direction and 76 ORFs (54%) were in a counter clockwise direction, according to the transcription orientation of the polyhedron gene.

### Comparison of HearMNPV ORFs to other baculoviruses

The overall gene arrangement and the homology between genes of the HearMNPV and other baculoviruses genomes were compared using Identity-GeneParity analysis [26]. The gene content and organization of HearMNPV were compared with a group I NPV (AcMNPV [32]), Group II NPVs (MacoNPV-B [33] MacoNPV-A [34], HearSNPV-G4 [7] and AgseNPV [35]), and GV (HearGV [11]. HearMNPV shares 117 ORFs with AcMNPV, 161 ORFs with MacoNPV-B, 159 ORFs with MacoNPV-A, 123 with HearSNPV-G4, 147 with AgseNPV, and 89 with HearGV. The average amino acid sequence identities between HearMNPV and AcMNPV, MacoNPV-B, MacoNPV-A, HearSNPV-G4, AgseNPV and HearGV were 44.7%, 98.5%, 90.2%, 41.0%, 58.5%, and 38.6%, respectively.

**Figure 4:**
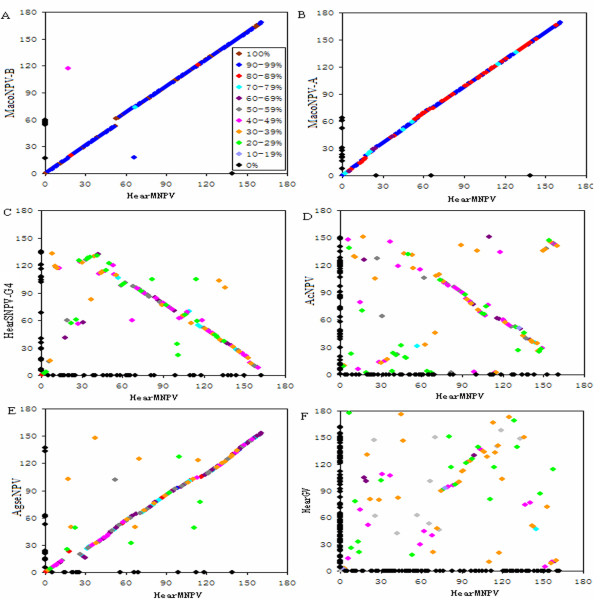
**Identity-Gene Parity Plots of HearMNPV with MacoNPV-B (A), MacoNPV-A (B), HearSNPV-G4 (C), AcMNPV (D), AgseNPV (E), and HearGV (F).** Amino acid identity (%) of individual homologous ORFs of HearMNPV compared to other baculoviruses are shown in various colors. ORFs unique to each virus are placed on the x-axis and y-axis, respectively (black diamonds).

Comparison of the gene order between HearMNPV and MacoNPV-B revealed a significantly gap between *orf 52* to *orf 53* in the HearMNPV genome. The gap corresponds to a region of MacoNPV-B comprising *orf54, 55, 56, 57, 58, 59,* and *60*. In addition, the *orf66* and *orf17* of HearMNPV are homologous to the *orf18* and *orf117* of MacoNPV-B with 99, 41.2% aa identity, respectively (Table 1). However, the locations of these homologues are not conserved. Relative to each other HearMNPV (*x-*axis) and MacoNPV-B (*y-*axis) contain 1 and 8 unique genes, respectively. However, HearMNPV and MacoNPV-B maintain perfect co-linearity in gene content and arrangement (Figure 4A).

The gene arrangement of HearMNPV was also completely collinear with that of MacoNPV-A. The result of the Identity-GeneParity analysis showed that relative to each other HearMNPV (*x-*axis) and MacoNPV-A (y-axis) contain 2 and 10 unique genes, respectively. There was also high collinearity between HearMNPV and MacoNPV-A (Figure 4B).

In terms of gene content, arrangement, and homology level, HearMNPV is significantly distant from HearSNPV-G4, although they infect the same host, *H. armigera*. Relative to each other HearMNPV (*x-*axis) and HearSNPV-G4 (y-axis) contain 38 and 18 unique genes, respectively, and these genes are distributed throughout the genomes (Figure 4C). The ‘left’ part of the HearMNPV genome (ORF5-69) displayed a high degree of gene scrambling in the gene parity plot analysis. The homologous ORFs from HearMNPV 70 to 160 are approximately collinear with the HearSNPV-G4 ORFs 8 to 96; however, the direction of the diagonal indicates these regions are inverted, relative to each other, except for HearMNPV ORF102-107 (corresponding to HearSNPV-G4 ORF62–67). (Figure 4C)

Relative to each other HearMNPV (*x-*axis) and AgseNPV (y-axis) contain 15 and 11unique genes, respectively. The collinearity between HearMNPV and AgseNPV was higher than that between HearMNPV and HearSNPV-G4, and lower than that between HearMNPV and MacoNPV-B or MacoNPV-A. (Figure 4E)

The collinearity between HearMNPV and AcMNPV from Group I was lower than those between HearMNPV and NPVs from group II (Figure 4D); the parity analysis of HearMNPV and HearGV ORFs displayed a much more dispersed pattern (Figure 4F).

### Phylogenetic analysis

**Figure 5:**
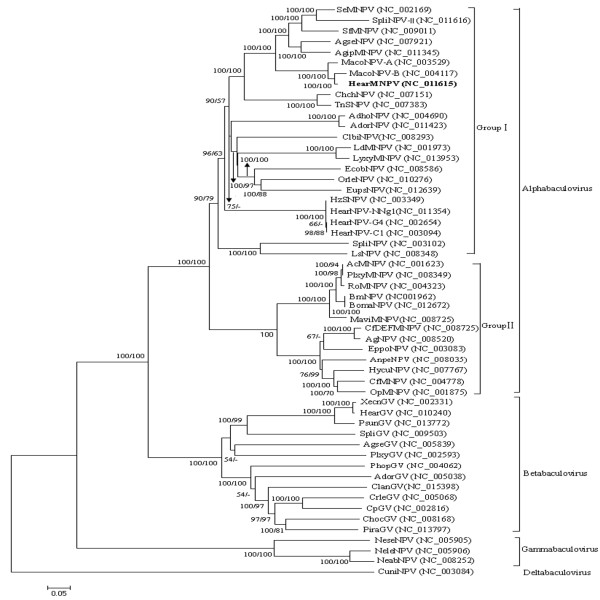
**Phylogenetic analysis of concatenated amino acid sequence alignments, showing bootstrap values >50% for NJ and MP trees at each node (NJ/MP).** The location of HearMNPV is shown in bold. The GenBank accession numbers of each virus are listed after the names.

Based on 29 concatenated, conserved genes [36], a phylogenetic tree was estimated for 54 baculoviruses. The results reflected the current systematic assignment of the viruses (Figure 5), indicating that HearMNPV and MacoNPV-B are grouped together and are distinct from HearSNPV-G4 HearSNPV-C1 and HearSNPV-NNg1. In addition, the phylogenetic analysis of three highly conserved genes (*lef-8*, *lef-9*, and *polh*) indicated that the HearMNPV sequences were separated from the other eighteen HearMNPV isolates [14]. These results imply that HearMNPV is a new isolate that differs from HearSNPV.

### Genomic Comparison between HearMNPV and MacoNPV-B: HearMNPV lacks a 5.4-kb fragment that contains five ORFs

**Figure 6:**
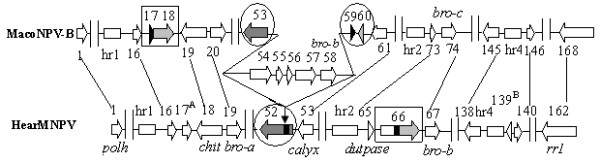
**Comparison of the genome structure of HearMNPV and MacoNPV-B.** The left and right arrows represent ORFs in HearMNPV and MacoNPV-B genomes, respectively. The numbers above the arrows represent the names of the ORFs in HearMNPV and MacoNPV-B genomes. The lines between the names of the ORFs represent homologies between the HearMNPV and MacoNPV-B genomes. The black region of arrows in the circle or box represent the nucleotide sequences homologies and the gray region of arrows in the circle or box represent the amino acid sequence homologies. The down arrow indicates the sites where the 5.4-kb fragment is inserted. The letter A indicates that the ORF of HearMNPV has no homolog in the corresponding position of MacoNPV-B. The letter B represents the ORF unique to HearMNPV. Double Vertical Lines represent ORFs that are not in the HearMNPV and MacoNPV-B genomes.

Compared with the MacoNPV-B genome, the HearMNPV genome does not have a 5.4-kb fragment that contains ORF54, 55, 56, 57, and 58 (Figure 6). The nucleotide identities between HearMNPV orf52 1–633 bp, 639–896 bp, and 853–1050 bp and MacoNPV-B *orf 53*, *orf 59*, *orf 60* are 98%, 98%, and 95%, respectively. Amino acids 147–349 of the protein encoded by HearMNPV *orf52* are 100% identical to those of the protein encoded by MacoNPV-B *orf 53* and amino acids (aa) 1–65 of the protein encoded by HearMNPV *orf52* are 86% identical to the amino acid sequence of MacoNPV-B *orf 60* (Figure 6, indicated by the gray parts in the circles and arrows). However, there was no aa sequence identity between the proteins encoded by HearMNPV *orf52* and MacoNPV-B *orf 59* (Figure 6, indicated by the black parts in the circles and arrows). The MacoNPV-A genome also lacked the 5.4-kb fragment, suggesting that an insertion in the genome might have lead to the division of ORF59 in MacoNPV-A [33].

According to the sequence analysis of 54 whole genomes of baculoviruses, the 5.4-kb fragment present in MacoNPV-B but not in HearMNPV shared homologous sequences with XecnGV [30] and HearGV [11], by reverse alignment (Table 2). However, this phenomenon was not observed in other genomes. Combined with the phylogenetic analysis (Figure 5), the results suggest that the 5.4-kb fragment was gained during evolution of MacoNPV-B and thus the common ancestor of HearMNPV ORF52 evolved to MacoNPV-B ORF53, ORF59, and ORF60 through gaining 5.4-kb fragment, together with subsequent nucleotide mutations, deletions, and insertions (Figure 6). For recombination to occur, the different viruses species have to be coinfecting the same host at the same time. A relatively recent recombination event between ancestors of MacoNPV-B and XecnGV resulted in the insertion of a 5.4-kb fragment from an ancestor of XecnGV into the genome of an ancestor of MacoNPV-B genome, suggesting that these lineages were capable of infecting the same host species at some point during their history [33]. HearMNPV and HearGV could infect the same host cotton bollworm, *H. armigera*, which provides the opportunity for the natural recombination between two viruses. However, HearMNPV did not gain the 5.4-kb fragment from HearGV by recombination.

**Table 2:** Comparison of ORFs aa identity from 5.4-kb fragment of MacoNPV-B, XecnGV and HearGV

**MacoNPV-B**	**Homologoues (% aa identity)**				
	**ORF54**	**ORF55**	**ORF56**	**ORF57**	**ORF58**
XecnGV	ORF65(98)	ORF64(98)	ORF62(93)	ORF61(98)	ORF131(84) ORF60(60)
HearGV	ORF60(94)	ORF59(94)	ORF58(55)	ORF57(94)	ORF133(89) ORF54(50)

### HearMNPV ORF66

The nucleotide sequence of ORF66 has high nucleotide sequence similarity to MacoNPV-B’s ORF17 and ORF18. Presumably, a mutation gave rise to the division of HearMNPV ORF66 into two open read frames in MacoNPV-B.

HearMNPV ORF66, located between ORF65 (*dutpase*) and ORF67 (*bro-b*), is 1779 bp long and encodes a protein 592aa. The aa sequence identity is 99% between the first 301aa of HearMNPV ORF66’s (874–1779 bp) and MacoNPV-B ORF18 (301aa). However, the genome sequence of HearMNPV ORF66 and MacoNPV-B ORF18 are not collinear (Figure 4A, Figure 6).

Nucleotides 676–968 of HearMNPV ORF66 are 95% identical to MacoNPV-B ORF17; however, the amino acids encoded by this nucleotide sequence did not share amino acid identity with the protein encoded by MacoNPV-B ORF17 because of frameshifts and other mutations of a few nucleotides. There is also no sequence similarity between the N-terminal region (1–675 bp) of HearMNPV ORF66 and MacoNPV-B, either at the nucleotide or amino acid level (shown in the boxes of Figure 6).

The HearMNPV ORF66 protein is 92% identical to the five homologous *hr1, hr2, hr3, hr4,* and *hr5*, each of approximately 608aa in size, of *Heliothis virescens* ascovirus -3e (HvAV-3e) [37], and 85% identical to the proteins encoded by *orf34* (564aa) and *orf77* (606aa) of *Spodoptera frugiperda* ascovirus-1a (SfAV-1a) [38]. The comparison showed that these homologous ORFs have four conserved cysteine domains, suggestive of a zinc-binding domain, hypothesized to be a DNA binding domain. This putative domain is found at the C terminus of a large number of transposase proteins, indicated that this might be related to gene duplication in the genome.

**Figure 7:**
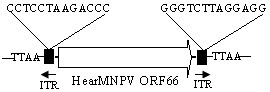
**Diagram of the region with two DNA sequences flanking a putative Transposase ORF (HearMNPV *orf66*) of 1 779 bp encoding a protein with 592 amino acids.** The tetranucleotide TTAA duplicated is characteristic of a transposition event by a transposable element. ITR represent the inverted terminal repeats.

Interestingly, we have found an element in the right and left flanking DNA sequences of HearMNPV *orf66* had two perfect inverted terminal repeats (ITRs) of 13 nucleotides. Moreover, the tetranucleotide5^′^-TTAA-3^′^, which is very common in transposition of the TTAA family, is duplicated upon this element [39,40] (Figure 7). This indicated that this element could insert exclusively at this insertion site (TTAA). However, the ORF66 has no amino acid identity with *piggyBac* transposase [41]by blastp analysis. Sequence analysis showed that the right and left flanking DNA sequences of HvAV-3e *hr1, hr2, hr3, hr4,* and *hr5* also have two perfect ITRs of 13 nucleotides. However, the left flanking DNA sequences of MacoNPVB *orf18* lacked the sequence CCTCCTAAGACCC. These results indicated homologous of HearMNPV orf66 in MacoNPVB was split into MacoNPVB *orf18* and *orf17* during evolution.

**Figure 8:**
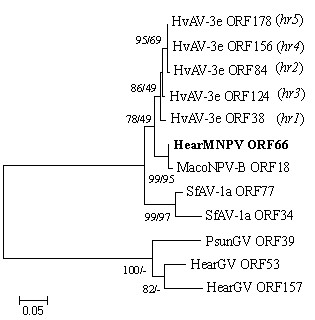
**Phylogenetic analysis of the HearMNPV ORF66 amino acid sequence.** The phylogenetic tree shows bootstrap values >50% for NJ and MP trees at each node (NJ/MP). The location of HearMNPV ORF66 is shown in bold. The sequences used are from *Mamestra configurata* NPV-96B (ORF18), *Helicoverpa armigera* GV(HearGV (ORF53 and ORF157), *Pseudaletia unipuncta* GV(ORF39), *Heliothis virescens* ascovirus 3e (HvAV-3e *hr1*–*hr5*), and *Spodoptera frugiperda* ascovirus 1a (SfAV-1a ORF34 and ORF77).

Searching for homologs of HearMNPV ORF66 among the baculoviruses revealed that only HearMNPV ORF66 (592aa), MacoNPV-B ORF18 (301aa), HearGV ORF53 (572aa), ORF157 (572aa), ORF157 (576aa), and PsunGV ORF39 (571aa) are homologous ORFs. The phylogenetic analysis indicated that HearGV ORF53, ORF157*,* and PsunGV ORF39 belong to the same phylogenetic branch, while HearMNPV ORF66, MacoNPV-B ORF18, HvAV-3e *hr1–hr5*, and SfAV-1a ORF34 and 37 belong to the same phylogenetic clade (Figure 8). HearMNPV and HvAV-3e are both isolated from cotton bollworms, HearMNPV ORF66 and HvAV-3e *hr1–hr5* share a flank structure, and have the highest amino acid identity among the homologous genes in baculovirus and ascoviridae to date (excluding unreleased relevant data). This data indicated that these genes might have been exchanged among species and genera.

The genomic differences between HearMNPV and MacoNPV-B are mainly located between *hr1* and *hr2*, including the deletion of the 5.4 kb fragment in HearMNPV and the changes in ORF66, both of which were close to a *bro* gene (Figure 6).

### HearMNPV ORF17

The locations of the HearMNPV ORF17 and its homologue in the MacoNPV-B genome are not conserved. HearMNPV ORF17 only has 41.2% aa identity to MacoNPV-B ORF117(e = 6e^-46^, with 98% query coverage), while HearMNPV ORF110 was collinear at an aa identity of 97.2% with MacoNPV-B ORF117, indicating that HearMNPV ORF17 has no significant collinearity with the homologous ORF of MacoNPV-B.

### HearMNPV unique ORF

HearMNPV ORF139 is 264 bp long and encodes a protein of 87 aa. There is an early promoter CATT motif in the 180 bp region upstream of the start codon. Using both BLASTX and BLASTP searching, no homologous protein was found among baculoviruses.

#### bro genes

The occurrence of the baculovirus repeat ORF (*bro*) gene family is a striking feature in many baculovirus genomes [42]. *bro* genes are associated with regions of viral genome rearrangement [43]. BmNPV BRO proteins have nucleic acid binding activity that influences host DNA replication and transcription [44]. BRO proteins function as nucleocytoplasmic shuttling proteins that utilize the CRM1-mediated nuclear export pathway [45]. We identified six bro genes dispersed among the genome of HearMNPV and named them *bro-a* to *bro-f*, according to the order of their appearance on the linearized genome. There are eight and seven *bro* genes in MacoNPV-A and MacoNPV-B, respectively. The *bro* genes are classified into four groups, based on the similarity of the 41-amimo acid core domain sequences used for LdMNPV BRO protein classification [46]. HearMNPV *bro-c*, *bro-d*, and *bro-e* belong to group I *bro* genes, *bro-a* and *bro-b* belong to group II *bro* genes, *bro-f* belongs to group IV There is no bro gene corresponding to MacoNPV-B *bro-b*, which belongs to group III. The HearMNPV genome also lacks homologs of the MacoNPV-A *bro-a* (group I) and *bro-c* (groupIII) genes.

The HearMNPV *bro-a, -b, -c, -d, -e, - f* genes showed aa identities of 83%, 77.5%, 98.3%, 89.6%, 97.4%, and98.8% to MacoNPV-B *bro-a, -c, -d, -e, -f, -g*, respectively. MacoNPV-B *bro-b* is located in the region of 5.4 kb fragment of MacoNPV-B, which is lack in the HearMNPV genome.

The HearMNPV *bro-a* gene had an N-terminal region from aa 1 to aa 134 with aa identities of 63% and 95% to MacoNPV-B *bro-a* and MacoNPV-A *bro-b*, respectively. The C-terminal region, from aa 135 to aa 331, has aa identities of 98% and 93% to MacoNPv-B *bro-a* and MacoNPV-A *bro-b,* respectively. This suggested that *bro-a* C-terminal regions are the highly conserved portions in these three virus genomes.

HearMNPV *bro-f* shows high homology to a hypothetical protein P20 [47] from *Leucania separata* NPV (LeseNPV) and MacoNPV-A *bro-h*, which both encode 179 aa proteins with amino acid identities of 95% and 98%, respectively. HearMNPV *bro-f* shows the highest homology to MacoNPV-B *bro-g*, with an amino acid identity of 98.8%. However, amino acids 1–17 of HearMNPV *bro-f* are not found in MacoNPV-B *bro-g.*

When comparing the *bro* genes of HearMNPV with MacoNPV-B, the lowest aa identity is between HearMNPV *bro-b* and MacoNPV-B *bro-c*, at 77.5%. The HearMNPV ORF66 gene is adjacent to HearMNPV *bro-b* and has changed much comparing with ORF17 and ORF18, which are closest to MacoNPV-B *bro-c.*

The differences between HearMNPV *bro-c, d,* and *e* and their homologs in MacoNPV-B represent minor nucleotide insertions, deletions, and substitutions.

The *bro* genes of HearMNPV differed from those of MacoNPV-B in both sequence and number, which indicated that the *bro* gene region is one of the most important in genomic variation of baculoviruses. The differences between HearMNPV and MacoNPV-B (the 5.4 kb fragment and the location of ORF66) were found in the vicinity of a *bro* gene. These differences indicated that *bro* gene might play a role in gene exchange, and, consequently, viral virulence and host range.

#### hrs

**Figure 9:**
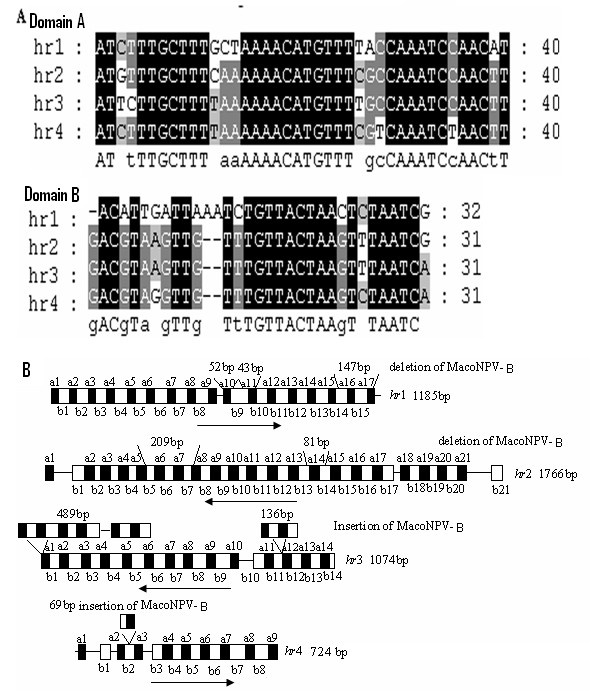
**Comparison of the *hr *regions between HearMNPV and MacoNPV-B.** Panel **A**: a deduced consensus sequence of domains A and B from each *hr* region was used for this alignment. Conserved sequences are indicated with different shading: black indicates 100% conservation, gray >70% conservation, and no shading <70% conservation. Panel **B**: arrows represent the direction and positions of the repeat-A and repeat-B regions, black boxes represent type A repeats and blank boxes represent type B repeats.

Variable numbers of *hr* sequences, composed of direct repeats containing a “core” imperfect palindrome and dispersed unevenly among the genome in AT rich intergenic regions, have been identified in most baculovirus genomes [48]. The baculovirus *hr*s act as enhancers of RNA polymerase II-mediated transcription of baculovirus early promoters [49], as well as functioning as origins of DNA replication in transient replication assays [50,51]. They are also sites of frequent recombinant and rearrangement in baculovirus genomes [52,53]. Four *hr*s were identified in the HearMNPV genome, with the sizes of 1185 bp (*hr1*), 1766 bp (*hr2*), 1074 bp *(hr3*) and 724 bp (*hr4*), respectively. The *hr*s are distributed throughout the HearMNPV genome: between *orf14* and *orf15, orf63* and *orf64*, *orf130* and *orf131*, and *orf138* and *orf139* for *hr* 1, 2, 3, and 4, respectively. Sequence analysis confirmed that the four *hr*s comprise two apparent domains with perfect or near-perfect 40 bp palindromes (designated type A) and 31 bp flanking repeats (designated type B) at the head/end of one or both sides of the palindromes (Figure 9A). Each *hr* repeat sequence comprises two apparent domains (type A and type B) that is similar to that described for MacoNPV-A and MacoNPV-B [33,34]. However, the repeat unit numbers in each *hr* of HearMNPV was different from MacoNPV-A and MacoNPV-B.

The four *hr*s of HearMNPV are located at similar positions in the genome as those of MacoNPV-B and MacoNPV-A. Sequence alignment between HearMNPV and MacoNPV-B *hr*s indicated that these four homologous regions had some insertions/deletions of different sizes, giving rise to identities of 92.0%, 92.3%, 86.6%, 81.9%, respectively. *hr1* has three insertions, two of 52 bp and 43 bp that contained only a type A repeat, and one of 147 bp that contained type A and type B repeats. *hr2* has two insertions (209 bp and 81 bp) that contained both type A and type B repeats. *hr3* has the biggest deletion (489 bp) and another large deletion of 136 bp, which also contained both type A and type B repeats. *hr4* has a small deletion of 69 bp that also contains both type A and type B repeats (Figure 9B). The HearMNPV *hr4* (724 bp) is shorter than the MacoNPV-B *hr4* (1178 bp) occurrence, probably caused by the presence of HearMNPV ORF139, which is adjacent to HearMNPV *hr4*. HearNPV NNg1 contains five *hr*s (hr1-hr5), similar to HearNPV C1, G4, and HzNPV. The arrangement of these *hr*s on the genome is almost the same in HearNPV C1, G4 and HzNPV, and it is possible that variability in the *hr* sequences affect not only progeny virus production, but also the insecticidal activity of the *Helicoverpa* spp. NPVs [9]. The homologous regions are also suggested to be responsible for the difference in virulence between two *Mamestra configurata* NPV-A variants, v90/4 and v90/2 [54], indicating that the difference in the organization of the homologous regions of HearMNPV and MacoNPV-B are possibly associated with mechanisms of recombination.

## Conclusion

HearMNPV differs significantly from HearSNPV not only in biological properties and morphology, but also in gene content, arrangement, and homology level based on genome sequence comparison, which considered to be different viruses, and not variants of the same virus. Although the average amino acid sequence identity between HearMNPV and MacoNPV-B is 98.5%, but their effective host range are different. Moreover, a 5.4-kb segment of the MacoNPV-B genome which is the apparent result of recombination with an ancestor of XecnGV is absent in the HearMNPV genome, suggesting that the recombination event responsible for the occurrence of this 5.4 kb segment occurred after the divergence of MacoNPV-B and HearMNPV. The location and length of HearMNPV *orf66* and MacoNPV-B *orf18* are different in their respective genomes. Phylogenetic analysis indicated that these events may occur after MacoNPV-B and MacoNPV-A separated from their ancestor. These distinct differences between HearMNPV and MacoNPV-B may account for their different host range.

## Competing interests

The authors declare that they have no competing interests.

## Authors’ contributions

PT carried out the molecular cloning and sequence analysis works and drafted the manuscript. HZ prepared the virus genomic library. YL, BH and GW participated in the sequence analysis and draft preparation. QQ and ZZ performed experiment design and paper writing. All authors read and approved the final manuscript.

## References

[B1] Zanotto PM Kessing BD Maruniak JE Phylogenetic interrelationships among baculoviruses: evolutionary rates and host associations J Invertebr Pathol 1993 62 147 164 10.1006/jipa.1993.1090 8228320

[B2] Jehle JA Blissard GW Bonning BC Cory JS Herniou EA Rohrmann GF Theilmann DA Thiem SM Vlak JM On the classification and nomenclature of baculoviruses: a proposal for revision Arch Virol 2006 151 1257 1266 10.1007/s00705-006-0763-6 16648963

[B3] Zalucki MP Murray DAH Gregg PC Fitt GP Twine PH Jones C Ecology of *Helicoverpa-Armigera* (Hubner) and *Heliothis-Punctigera* (Wallengren) in the Inland of Australia - Larval Sampling and Host-Plant Relationships During Winter and Spring Aust J Zool 1994 42 329 346 10.1071/ZO9940329

[B4] Moscardi F Assessment of the application of baculoviruses for control of Lepidoptera Annu Rev Entomol 1999 44 257 289 10.1146/annurev.ento.44.1.257 15012374

[B5] Shieh TR Industrial production of viral pesticides Adv Virus Res 1989 36 315 343 2660496 10.1016/s0065-3527(08)60589-8

[B6] Zhang G Research, development and application of *Heliothis* viral pesticide in China Resour Environ Yangtze Valley 1994 3 39 44

[B7] Chen X IJkel WF Tarchini R Sun X Sandbrink H Wang H Peters S Zuidema D Lankhorst RK Vlak JM The sequence of the *Helicoverpa armigera* single nucleocapsid nucleopolyhedrovirus genome J Gen Virol 2001 82 241 257 11125177 10.1099/0022-1317-82-1-241

[B8] Zhang CX Ma XC Guo ZJ Comparison of the complete genome sequence between C1 and G4 isolates of the *Helicoverpa armigera* single nucleocapsid nucleopolyhedrovirus Virology 2005 333 190 199 10.1016/j.virol.2004.12.028 15708604

[B9] Ogembo JG Caoili BL Shikata M Chaeychomsri S Kobayashi M Ikeda M Comparative genomic sequence analysis of novel *Helicoverpa armigera* nucleopolyhedrovirus (NPV) isolated from Kenya and three other previously sequenced *Helicoverpa* spp NPVs Virus Genes 2009 39 261 272 10.1007/s11262-009-0389-3 19634008

[B10] Chen X Zhang WJ Wong J Chun G Lu A McCutchen BF Presnail JK Herrmann R Dolan M Tingey S Comparative analysis of the complete genome sequences of *Helicoverpa zea* and *Helicoverpa armigera* single-nucleocapsid nucleopolyhedroviruses J Gen Virol 2002 83 673 684 11842262 10.1099/0022-1317-83-3-673

[B11] Harrison RL Popham HJ Genomic sequence analysis of a granulovirus isolated from the Old World bollworm, *Helicoverpa armigera* Virus Genes 2008 36 565 581 10.1007/s11262-008-0218-0 18418706

[B12] Pritchett DW Scott HA Young SY Serological relationships of five nuclearpolyhedrosis viruses from lepidopterous species J Invertebr Pathol 1979 33 2 183 188 10.1016/0022-2011(79)90151-4

[B13] Gettig RR McCarthy WJ Genotypic variation among wild isolates of *Heliothis* spp nuclear polyhedrosis viruses from different geographical regions Virology 1982 117 1 245 252 10.1016/0042-6822(82)90523-2 18635119

[B14] Rowley DL Popham HJ Harrison RL Genetic variation and virulence of nucleopolyhedroviruses isolated worldwide from the heliothine pests *Helicoverpa armigera*, *Helicoverpa zea*, and *Heliothis virescens* J Invertebr Pathol 2011 107 2 112 126 10.1016/j.jip.2011.03.007 21439295

[B15] Chen Y Lin X Yi Y Lu Y Zhang Z Construction and application of a baculovirus genomic library Z Naturforsch C 2009 64 574 580 19791511 10.1515/znc-2009-7-817

[B16] Nie ZM Zhang ZF Wang D He PA Jiang CY Song L Chen F Xu J Yang L Yu LL Complete sequence and organization of *Antheraea pernyi* nucleopolyhedrovirus, a dr-rich baculovirus BMC Genomics 2007 8 248 10.1186/1471-2164-8-248 17650316 PMC1976136

[B17] Wang HZ Huang Y Si YH Fang MG Cheng XW Vlak JM Hu ZH A Novel Expression System of *Helicoverpa armigera* single-nucleocapsid nucleopolydrovirus Virologica Sinica 2002 17 319 325

[B18] Wang H Deng F Pijlman GP Chen X Sun X Vlak JM Hu Z Cloning of biologically active genomes from a *Helicoverpa armigera* single-nucleocapsid nucleopolyhedrovirus isolate by using a bacterial artificial chromosome Virus Res 2003 97 57 63 10.1016/j.virusres.2003.07.001 14602197

[B19] Wu D Deng F Sun X Wang H Yuan L Vlak JM Hu Z Functional analysis of FP25K of *Helicoverpa armigera* single nucleocapsid nucleopolyhedrovirus J Gen Virol 2005 86 9 243924 243944 10.1099/vir.0.81110-0 16099901

[B20] Zheng GL Li CY Zhou HX Li SW Li GX Xue M Establishment of two new cell lines from the embryonic tissue of *Helicoverpa armigera* (Lepidoptera:Noctuidae)and their responses to baculovirus infection Acta Entom ologica Sinica 2010 53 167 174

[B21] Mangé A Prudhomme JC Comparison of *Bombyx mori* and *Helicoverpa armigera* cytoplasmic actin genes provides clues to the evolution of actin genes in insects Mol Biol Evol 1999 16 165 172 10.1093/oxfordjournals.molbev.a026099 10028284

[B22] Wheeler DL Church DM Federhen S Lash AE Madden TL Pontius JU Schuler GD Schriml LM Sequeira E Tatusova TA Database resources of the National Center for Biotechnology Nucleic Acids Res 2003 31 28 33 10.1093/nar/gkg033 12519941 PMC165480

[B23] Kool M Vlak JM The structural and functional organization of the *Autographa californica* nuclear polyhedrosis virus genome Arch Virol 1993 130 1 16 10.1007/BF01318992 8389114

[B24] Altschul SF Gish W Miller W Myers EW Lipman DJ Basic local alignment search tool J Mol Biol 1990 215 403 410 2231712 10.1016/S0022-2836(05)80360-2

[B25] Lauzon HA Jamieson PB Krell PJ Arif BM Gene organization and sequencing of the *Choristoneura fumiferana* defective nucleopolyhedrovirus genome J Gen Virol 2005 86 945 961 10.1099/vir.0.80489-0 15784888

[B26] Hu ZH Arif BM Jin F Martens JW Chen XW Sun JS Zuidema D Goldbach RW Vlak JM Distinct gene arrangement in the *Buzura suppressaria* single-nucleocapsid nucleopolyhedrovirus genome J Gen Virol 1998 79 2841 2851 9820162 10.1099/0022-1317-79-11-2841

[B27] Tamura K Peterson D Peterson N Stecher G Nei M Kumar S MEGA5: molecular evolutionary genetics analysis using maximum likelihood, evolutionary distance, and maximum parsimony methods Mol Biol Evol 2011 28 2731 2739 10.1093/molbev/msr121 21546353 PMC3203626

[B28] Asser-Kaiser S Fritsch E Undorf-Spahn K Kienzle J Eberle KE Gund NA Reineke A Zebitz CP Heckel DG Huber J Rapid emergence of baculovirus resistance in codling moth due to dominant, sex-linked inheritance Science 2007 317 1916 1918 10.1126/science.1146542 17901332

[B29] Lu HS Insects virus and virus disease of insect 1982 Beijing: Science Press

[B30] Hayakawa T Ko R Okano K Seong SI Goto C Maeda S Sequence analysis of the *Xestia c-nigrum* granulovirus genome Virology 1999 262 277 297 10.1006/viro.1999.9894 10502508

[B31] IJkel WF van Strien EA Heldens JG Broer R Zuidema D Goldbach RW Vlak JM Sequence and organization of the *Spodoptera exigua* multicapsid nucleopolyhedrovirus genome J Gen Virol 1999 80 3289 3304 10567663 10.1099/0022-1317-80-12-3289

[B32] Ayres MD Howard SC Kuzio J Lopez-Ferber M Possee RD The complete DNA sequence of *Autographa californica* nuclear polyhedrosis virus Virology 1994 202 586 605 10.1006/viro.1994.1380 8030224

[B33] Li L Donly C Li Q Willis LG Keddie BA Erlandson MA Theilmann DA Identification and genomic analysis of a second species of nucleopolyhedrovirus isolated from *Mamestra configurata* Virology 2002 297 226 244 10.1006/viro.2002.1411 12083822

[B34] Li Q Donly C Li L Willis LG Theilmann DA Erlandson M Sequence and organization of the *Mamestra configurata* nucleopolyhedrovirus genome Virology 2002 294 106 121 10.1006/viro.2001.1313 11886270

[B35] Jakubowska AK Peters SA Ziemnicka J Vlak JM van Oers MM Genome sequence of an enhancin gene-rich nucleopolyhedrovirus (NPV) from *Agrotis segetum*: collinearity with *Spodoptera exigua* multiple NPV J Gen Virol 2006 87 537 551 10.1099/vir.0.81461-0 16476975

[B36] van Oers MM Vlak JM Baculovirus genomics Curr Drug Targets 2007 8 1051 1068 10.2174/138945007782151333 17979665

[B37] Asgari S Davis J Wood D Wilson P McGrath A Sequence and organization of the *Heliothis virescens* ascovirus genome J Gen Virol 2007 88 1120 1132 10.1099/vir.0.82651-0 17374755

[B38] Bideshi DK Demattei MV Rouleux-Bonnin F Stasiak K Tan Y Bigot S Bigot Y Federici BA Genomic sequence of *Spodoptera frugiperda* Ascovirus 1a, an enveloped, double-stranded DNA insect virus that manipulates apoptosis for viral reproduction J Virol 2006 80 11791 11805 10.1128/JVI.01639-06 16987980 PMC1642580

[B39] Cary LC Goebel M Corsaro BG Wang HG Rosen E Fraser MJ Transposon mutagenesis of baculoviruses: analysis of *Trichoplusia ni* transposon IFP2 insertions within the FP-locus of nuclear polyhedrosis viruses Virology 1989 172 156 169 10.1016/0042-6822(89)90117-7 2549707

[B40] Wang J Miller ED Simmons GS Miller TA Tabashnik BE Park Y piggyBac-like elements in the pink bollworm, *Pectinophora gossypiella* Insect Mol Biol 2010 19 177 184 10.1111/j.1365-2583.2009.00964.x 20017756

[B41] Carpes MP Nunes JF Sampaio TL Castro ME Zanotto PM Ribeiro BM Molecular analysis of a mutant *Anticarsia gemmatalis* multiple nucleopolyhedrovirus (AgMNPV) shows an interruption of an inhibitor of apoptosis gene (iap-3) by a new class-II piggyBac-related insect transposon Insect Mol Biol 2009 18 6 747 757 10.1111/j.1365-2583.2009.00917.x 19788700

[B42] Bideshi DK Renault S Stasiak K Federici BA Bigot Y Phylogenetic analysis and possible function of bro-like genes, a multigene family widespread among large double-stranded DNA viruses of invertebrates and bacteria J Gen Virol 2003 84 2531 2544 10.1099/vir.0.19256-0 12917475

[B43] Willis LG Seipp R Stewart TM Erlandson MA Theilmann DA Sequence analysis of the complete genome of *Trichoplusia ni* single nucleopolyhedrovirus and the identification of a baculoviral photolyase gene Virology 2005 338 209 226 10.1016/j.virol.2005.04.041 15951000

[B44] Zemskov EA Kang W Maeda S Evidence for nucleic acid binding ability and nucleosome association of *Bombyx mori* nucleopolyhedrovirus BRO proteins J Virol 2000 74 6784 6789 10.1128/JVI.74.15.6784-6789.2000 10888617 PMC112195

[B45] Kang W Kurihara M Matsumoto S The BRO proteins of *Bombyx mori* nucleopolyhedrovirus are nucleocytoplasmic shuttling proteins that utilize the CRM1-mediated nuclear export pathway Virology 2006 350 184 191 10.1016/j.virol.2006.01.008 16483627

[B46] Kuzio J Pearson MN Harwood SH Funk CJ Evans JT Slavicek JM Rohrmann GF Sequence and analysis of the genome of a baculovirus pathogenic for *Lymantria dispar* Virology 1999 253 17 34 10.1006/viro.1998.9469 9887315

[B47] Xiao H Qi Y Genome sequence of *Leucania seperata* nucleopolyhedrovirus Virus Genes 2007 35 845 856 10.1007/s11262-007-0106-z 17763934

[B48] Kool M Ahrens CH Vlak JM Rohrmann GF Replication of baculovirus DNA J Gen Virol 1995 76 2103 2118 10.1099/0022-1317-76-9-2103 7561748

[B49] Theilmann DA Stewart S Tandemly repeated sequence at the 3' end of the IE-2 gene of the baculovirus *Orgyia pseudotsugata* multicapsid nuclear polyhedrosis virus is an enhancer element Virology 1992 187 97 106 10.1016/0042-6822(92)90298-4 1736547

[B50] Ahrens CH Pearson MN Rohrmann GF Identification and characterization of a second putative origin of DNA replication in a baculovirus of *Orgyia pseudotsugata* Virology 1995 207 572 576 10.1006/viro.1995.1119 7886962

[B51] Pearson MN Rohrmann GF *Lymantria dispar* nuclear polyhedrosis virus homologous regions: characterization of their ability to function as replication origins J Virol 1995 69 213 221 7983712 10.1128/jvi.69.1.213-221.1995 PMC188566

[B52] Hayakawa T Rohrmann GF Hashimoto Y Patterns of genome organization and content in lepidopteran baculoviruses Virology 2000 278 1 12 10.1006/viro.2000.0668 11112474

[B53] Harrison RL Bonning BC Comparative analysis of the genomes of Rachiplusia ou and *Autographa californica* multiple nucleopolyhedroviruses J Gen Virol 2003 84 1827 1842 10.1099/vir.0.19146-0 12810877

[B54] Li L Li Q Willis LG Erlandson M Theilmann DA Donly C Complete comparative genomic analysis of two field isolates of *Mamestra configurata* nucleopolyhedrovirus-A J Gen Virol 2005 86 91 105 10.1099/vir.0.80488-0 15604435

